# Books and Authors

**Published:** 1911-12

**Authors:** 


### BOOKS AND AUTHORS

**The Principles and Practice of Bandaging, by Gwilym G. Davis,** Philadelphia. Published by P. Blakiston's Son & Co., Philadelphia, 1911. 128 pages, illustrated with 164 cuts, $1.00.)

This is a very complete, systematic and compact treatise, well printed. As there are few controversial points and little opportunity for originality in this branch of surgery, comment is unnecessary. The only suggestion that we can make in the nature of a criticism is that for the use of medical students, it would be a little more convenient to have such a book of pocket size.

**Surgical Applied Anatomy.** By **Sir Frederick Treves,** F.R.C.S., Sergeant-Surgeon to H. M. the King, Late Lecturer on Anatomy at the London Hospital. New (6th) edition, thoroughly revised. Pocket size, 12mo, 676 pages, 137 illustrations, of which many are in colors. Cloth, red edges, $2.50, net. Lea & Febiger, Philadelphia and New York. 1911.

This familiar little red book, has been revised by Arthur Keith, M.D., LL.D., but retains the graphic style of the original author. It is so well and favorably known in earlier editions that we need only say that it is what it purports to be and that it leaves practically no room for a rival in the same field.

**A Manual of Pathology and Morbid Anatomy,** by **T. Henry Green,** M.D., F.R.C.P., London; eleventh edition, revised and enlarged by **W. Cecil Bosanquet,** M.A., M.D., F.R.C.P., London. Lea & Febiger, Philadelphia and New York, 1911. 642 pages, 350 illustrations, $4.50.

Forty years have elapsed since the first edition appeared. The history of scientific pathology is epitomized in the history of this standard text book. The book and the science have grown together. Its author has not only recorded but helped to make the advances of the science.

**Recipes,** compiled by **Clara S. Lord,** 1321 M. St., N. Y., Washington, D.C. 96 pages, paper covers, for sale by the author, 50 cents, post paid.

We review this book hastily as a glance through its pages gives an appetite that must be satisfied. It includes, but is by no means limited to the broths and pastes so often considered appropriate for the sick and convalescent. Miss Lord has evidently grasped the fact that a person in need of careful feeding needs nutriment and that nutriment means the use of ordinary food stuffs, properly prepared. It would not be advisable to turn the book over to a nurse to choose at random from its pages for every convalescent; it would be even less advisable to allow carte blanche for an acute case as of typhoid. Many of the recipes would be appropriate for persons in health and the general idea of the book is to afford a wide range of dainties, dainty ways of serving substantials, and novelties, out of which a discriminating choice may be made for those whose appetite needs stimulation.

---

**Lippincott's New Medical Dictionary,** second edition, 1911. Edited by **Henry W. Cattell,** A.M., M.D., J. B. Lippincott Co., Philadelphia. 1100 pages, freely illustrated, limp leather, thumb index, $5.00.

This is smaller than the usual large dictionary, due to the use of tough, thin paper, clear but rather small type and judicious condensation. A novel feature is the inclusion of biographic notes and portraits. While much collateral information is given, the editor has wisely stopped short of the attempt at a cyclopaedia. In the explanatory notes, we read that "There are not a few medical terms which are formed contrary to scientific principles of word formation. These errors, where they occur, are pointed out and the correct forms are given." However, there is no disposition to split hairs, and the dictionary is of things as they are rather than as they ought to be. For example, while the word *appendicitis* is properly criticized, *penischisis* appears without an apology and, while we find the correct term *nesteostomy,* in its proper alphabetic place, there is no reference to it under *jejunostomy,* where it is really needed as so few writers use the correct form, that most physicians would have difficulty in finding it.

These are minor defects, if indeed they are really defects at all in a dictionary, which should record actual usage rather than ideals.

---

**Electricity, Medical and Surgical,** by **Dr. Charles S. Potts** of Philadelphia. Published by Lea & Febiger, Philadelphia and New York, 1911. 509 pages, 356 illustrations and 6 plates; $

Section 1, on the general physical principles of electricity is written by Horace Clark Richards, Ph. D. and, while freed from technicalities, affords a clear conception of the general theory. Section 7, on the *x*-rays, is contributed by Dr. Henry K. Pancoast. The intervening sections cover, in a thorough manner, the physiologic effects, and diagnostic and therapeutic uses of the various electric currents. If we were asked to point out the special points of superiority of this work over others, we should answer: the uniform clearness of typography and illustration, to be credited to the publishers, and the avoidance by the author of the tendency to concentrate on some of the newer applications of electricity and to make it a mere picture gallery of *x*-ray investigations. In other words, it does ample justice to methods no longer novel but deserving retention and aims to furnish the physician with a complete working manual.

**Manual of Pathology,** including Bacteriology, the Technic of Post Mortems and Methods of Pathologic Research, by **W. M. Late Coplin,** M.D., Philadelphia. Published by P. Blackiston's Son & Co., Philadelphia, 1911. Fifth edition, 1139 pages, 612 illustrations and 12 plates, 11 colored. $4.50.

In reviewing so large a work, it is impossible to call attention to its excellence in detail. The author's and the publishers' reputations stand behind it.

---

**Currents of High Potential of High and Other Frequencies.** Second edition. By **William Benham Snow,** M.D. Author of "A Manual of Electro-Static Modes of Application, Therapeutics, Radiography and Radiotherapy," "Therapeutics of Radiant Light and Heat and Convective Heat," Editor of the Journal of Advanced Therapeutics, late Instructor in Electro-Therapeutics in the N. Y. Post-Graduate School and Hospital, etc. Published by the Scientific Authors' Publishing Co., 329 West 57th St., New York. Price, $3.00 net.

This work has been entirely revised, rewritten and enlarged. Forty cuts have been added, and the chapters on High Frequency Currents and Therapeutics have been revised and entirely rewritten. The work contains the results of the author's personal researches and investigations, and includes most that is valuable on the subject of High Potential Currents. The developments in the subject of Hypertension and its treatment by the d'Arsonval current, as well as the employment of direct d'Arsonvalization in the treatment of infection, has been entirely considered in this edition.

---

**The Origin of Life,** being an account of Experiments with Certain Superheated Saline Solutions in Hermetically Sealed Vessels, by **H. Charlton Bastian,** M.D., F.R.S., London. Published by G. P. Putnam's Sons, New York and London, 1911. 118 pages, 10 photo-micrographic plates, $1.50. This is one of the Science Series, prepared in such a way as not to be popular in the ordinary sense and yet sufficiently free from technicalities to appeal to any intelligent student of nature.

The author makes a strong plea for the spontaneous development of the simpler forms of life, both on the evidence of experiment and on the probability that evolution from geologically ancient bacteria would, by this time, have resulted in marked deviation from the original types. He also raises the question as to why we should admit, as we must, the development of living forms at some distant period and deny it for the present. Such a question is unanswerable for the evolutionist of extreme type, and, on reflection, equally so for the man who accepts creation in the limited personal sense. The scientific world has scarcely had time to recover from the overwhelming surprise at the results of such experiments, to view them without bias.

In this connection, it may not be out of place to call attention to the fact that negative results of certain experiments in regard to spontaneous generation, as well as the apparent lethal influence of pure water on living organisms may be due to minute quantities of copper, etc., in distilled water. As one professor in charge of a biologic department of a university said in reply to a question as to the principal line of investigation of his assistants, the main immediate issue seems to be to produce a pure $H_2O$.

---

**Sex in Relation to Society,** Vol. VI. Studies in Psychology of Sex. By **Havelock Ellis,** F. A. Davis Co., Philadelphia, 1911. 656 pages. Cloth, $3.00.

The publication of this volume, the last in the series of studies in the Psychology of Sex, completes thirty years of study on this important subject, by the author, who ranks among the foremost authorities on this question. The first five volumes previously reviewed in the BUFFALO MEDICAL JOURNAL at the time of publication of each, treat of the various forms of expression of the sexual impulse as experienced subjectively in the individual without regard to his surroundings or associations. Naturally there has been much that is unpleasant and even repulsive in the kinds of sexual inversions discussed. In fact, the publication of the first volume years ago in England, lead to the social ostracism of the author and the prohibition of its distribution in England, so that Ellis was obliged to print the remaining five volumes, the present included, in America and Germany.

But whatever criticism may be justly or unjustly made of these volumes, the present one deserves commendation. It treats essentially of sexual man in relation to society and discusses clearly the problems of sex in the space of chapters where other authors require volumes. The essential topics treated of are: The mother and the child, sexual education, the valuation of sexual love, the function of chastity, the problem of sexual abstinence, protitution, the conquest of venereal disease, marriage and the science of procreation—to none of which exception should be taken if read in the scientific spirit in which the work is written.

Whatever our own individual opinion may be of the author or his work, there is no question of its place in the literature of sex psychology. Physicians, as well as clergymen, who have these problems confronting them, could profitably peruse the book, even though there may be serious objection as to placing it in the hands of the average layman.

This volume, as well as its forerunners, can be purchased separately.

**Anders' and Boston's Medical Diagnosis.** Octavo of 1175 pages, with 443 illustrations. By **James M. Anders,** M.D., Ph.D., LL.D., Professor of the Theory and Practice of Medicine and of Clinical Medicine, Medico-Chirurgical College, Philadelphia; and **L. Napoleon Boston,** A.M., M.D., Adjunct Professor of Medicine, Medico-Chirurgical College, Philadelphia. W. B. Saunder's Co., Philadelphia. Cloth, $6.00 net; half morocco $7.50 net.

This volume is a carefully prepared scientific treatise on the important subject of Medical Diagnosis. It is written in collaboration by one of America's leading clinicians and a laboratory worker of known worth. Consequently, both the clinical features and laboratory methods are equally emphasized. To the older practitioner accustomed to give undue prominence to the distinctly clinical side of a case and the more recent graduate apt to err on the laboratory diagnosis, the work is equally useful as it acts as a check on the conservatism of the former and the enthusiasm of the latter.

The method in which each division is treated is both rational and practical. Particularly advantageous are the "Summary of Diagnostic Features, the Laboratory Findings and Differential Diagnosis," all of which are stated clearly, concisely and as succinctly as the work will permit. Moreover, the book is fully illustrated, there being numerous plates—some in color, all of which will be useful to emphasize and illustrate the special point or feature which it demonstrates.

The section on Nervous Diseases covers about 200 pages and is from the pen of Dr. Thomas H. Weisenburg, a well known neurologist of Philadelphia. In general treatment, this section conforms to the use of the work. Special mention should be made of the moving pictures which portray the tabetic gait in Locomotor Ataxia, the spastic gait in lateral sclerosis, the attitude and tremor in Parkinson's Disease, the tremor in multiple sclerosis, etc. This is a new feature in pictorial presentation in medical books and will be found very useful.

After careful perusal of the work, the reviewer is perfectly frank in stating that he finds everything to commend and nothing to criticize in this work. The book surely fills its place in medical literature and should prove indispensable to the student and practitioner.

---

**A Mother's Guide.** A Manual for the Guidance of mothers and nurses by **Francis Tweddell,** M.D., New York. Published by **James T. Daughterty,** 411 W. 59th St., New York City; 190 pages, $1.00.

This is a very handy little compend, by no means unworthy of the physician's attention. It is indexed and marginal headings facilitate reference. It may well be advised for the guidance of mothers, with such marking of chapters and verbal cautions as the particular case requires.

---

**Case Histories in Neurology, E. W. Taylor,** A.M., M.D., Boston. Published by W. M. Leonard, Boston. 305 pages. $3.00.

We have recently reviewed another book in this series, calling attention to the general excellence of the plan of systematic clinical presentation of facts. The present book gives a brief resume of diagnostic methods and types of paralysis. The first 23 cases described cover diseases of the peripheral nerves—mainly, of course, designated neuritis. Section 2, through case 58, deals with the spinal cord; section 3, through case 91, with the brain, including hemiplegias, tumors, abscess, syphilis, etc.; section 4, through cases 109 includes diseases of vague or undetermined pathologic basis, such as chorea, paralysis agitans, myxoedema, Meniere's disease, etc.; section 5 treats of psychoneuroses.

The cases are selected not so much with the idea of presenting types, as with that of illustrating by actual examples, problems in differential diagnosis, therapeutics, etc., liable to occur in anyone's practice and affording difficulties beyond the preparation of elementary treatises. At the same time, care is taken to illustrate types and not merely to exhibit an array of rare cases. Careful reading of such a work gives exactly the form of mental gymnastics that is requisite to develop professional strength of judgment.

---

The Fourth Physician, by Montgomery B. Pickett, A. C. McClurg & Co., Chicago, 1911; 144 pages, several illustrations in color. $1.00.

This is a pretty Christmas story, telling of the change of heart of an ambitious and rather mercenary young physician who lost—in both senses—just the rare case which he was most anxious to get to try his new method. It is especially designed for a gift book and, between the lines, we read the high estimate which the author has of the average physician.

---

**Manual of Physiology,** for students and practitioners, by **H. Willoughby Lyle,** M.D., B.S., F.R.C.S., London. Published by Oxford University Press, London, 1911. 750 pages, 1 colored plate and 135 figures. $4.00.

The now quite lengthy list of ferments, the less vague description of the haemopoietic organs, the references to hormones, the inclusion of a number of facts derived from clinical demonstration and careful, practical, experiments as well as from chemistry and microscopy, in place of former theories are a good example of the progress of physiology. The practitioner of medicine cannot as a rule take time to read prolix and abstruse literature but he certainly should read such a book as this, every few years. To many, it will be a surprise to learn how practical physiology has become and what a wealth there now is of definite knowledge

---

The Practitioners Visiting List for 1912. An invaluable pocket-sized book containing memoranda and data important for every physician, and ruled blanks for recording every detail of practice. The Weekly, Monthly and 30-Patient Perpetual contain 32 pages of data and 160 pages of classified blanks. The 60-Patient Perpetual consists of 256 pages of blanks alone. Each in one wallet-shaped book, bound in flexible leather, with flap and pocket, pencil with rubber, and calendar for two years. Price by mail, postpaid, to any address, $1.25. Thumb-letter index, 25 cents extra. Descriptive circular showing the several styles sent on request. Lea & Febiger, Publishers, Philadelphia and New York.

---

The Medical Record Visiting List for 1912, $1.50 and upward, according to size and binding. Wm. Wood & Co., New York. This pocket and desk companion has become so well established as to require little in the way of comment. There are the usual inclusions of dosage, treatment of poisoning and other emergency cases, obstetric tables and the like. In these days in which the economic side of practice has become so important, we need not emphasize the value of an immediate, careful entry of professional services, nor the fact that this book reduces such labor to the minimum.

The American Illustrated Medical Dictionary, 6th revised edition by Dr. W. A. Newman Dorland; published by the W. B. Saunders Co., Philadelphia, 1911. 986 pages, 323 illustrations, 119 in colors, $4.50 without; $5 with thumb index, flexible leather. This edition contains over 7000 new terms. A convenient detail is the capitalization of proper names only. Every word is pronounced and defined exactly without reference to another heading. Phrases are explained under the corresponding nouns. Anatomic and analogous tables are given so that the reader obtains not merely a definition but very complete information on a subject. Veterinary and dental terms are included.

